# Benign Subcutaneous Emphysema of the Upper Limb: A Case Report

**DOI:** 10.5704/MOJ.1611.002

**Published:** 2016-11

**Authors:** SM Rabiul Islam, KG Mamman, KC Pande

**Affiliations:** Department of Orthopaedic Surgery, Raja Isteri Pengiran Anak Saleha (RIPAS) Hospital, Bandar Seri Begawan, Brunei Darussalam

**Keywords:** Subcutaneous emphysema, upper limb, gas gangrene, necrotising fasciitis

## Abstract

Subcutaneous emphysema is the presence of gas or air in the subcutaneous tissue plane. The term is generally used to describe any soft tissue emphysema of the body wall or limbs, it can result from benign causes, most commonly secondary to trauma or from a life-threatening infection by gas gangrene or necrotising fasciitis. A case of subcutaneous emphysema involving the upper limb resulting from a trivial laceration to the elbow is reported and the importance of distinguishing between the two causes of subcutaneous emphysema is highlighted.

## Introduction

Benign subcutaneous emphysema of the upper limb may result from a number of causes^1–3^. It should be differentiated from gas in the soft tissues resulting from other life threatening conditions as the treatment and outcome of the two differ widely. The following case highlights the importance of careful history, clinical and laboratory examinations to differentiate between the two conditions.

## Case Report

A healthy 49 years old right-handed woman presented to the Accident and Emergency Department in a district hospital with history of fall and sustaining a trivial laceration to the right olecranon region. The wound was irrigated and cleaned and closed with adhesive skin strips. She was discharged with analgesia without any radiological evaluation.

Five to six hours later the patient came back with extensive swelling of the right forearm. Radiograph of the right forearm, revealed subcutaneous emphysema from distal arm to the dorsum of wrist. She was referred to our tertiary hospital for further evaluation and management.

**Fig. 1:**
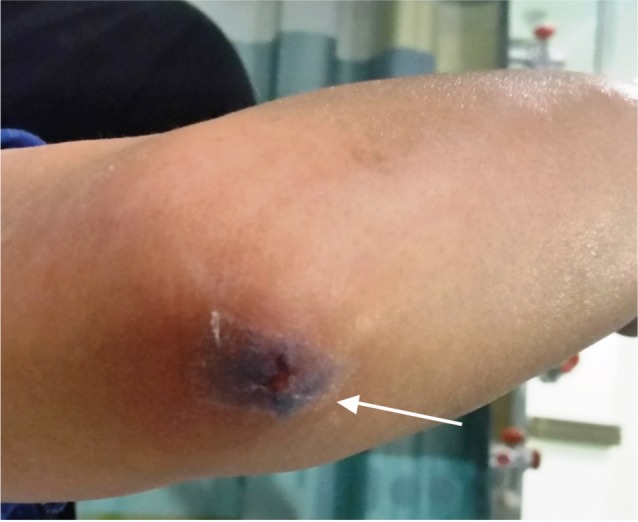
Photograph showing laceration over the olecranon with swelling of the forearm due to subcutaneous emphysema.

**Fig. 2:**
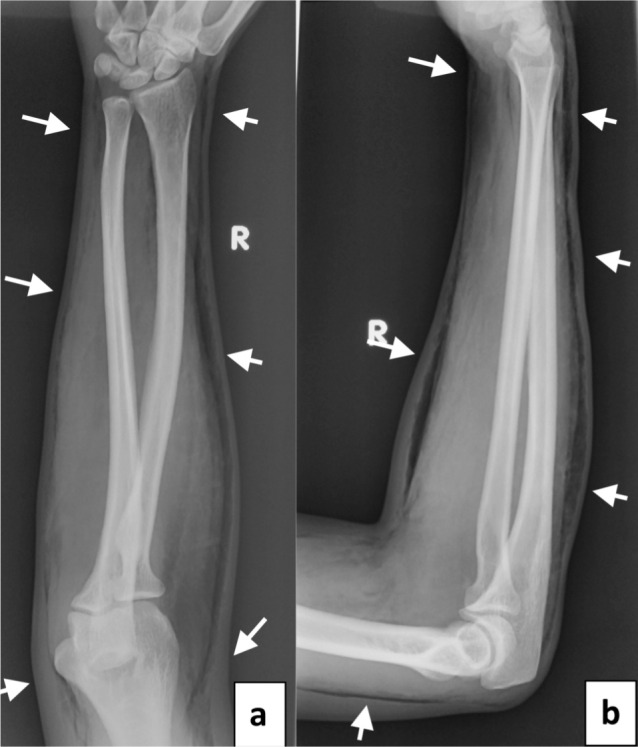
a and b: Antero-posterior and lateral radiograph of the right forearm showing subcutaneous emphysema limited to the subcutaneous plane, extending to the dorsum of hand and posterior aspect of arm.

On examination in the tertiary hospital, the patient had localised redness at the wound site over the olecranon (Fig. 1). Crepitus was palpable throughout the forearm on both flexor and extensor compartments extending proximally into the distal arm and distally onto the dorsal aspect of wrist. Radiographs of the forearm and arm revealed air in the subcutaneous tissues of the right lower arm, whole forearm and dorsal aspect of the wrist (Fig. 2a and b). The patient was afebrile with stable vital signs. There was no evidence of infection on the blood tests with normal white cell count and C-reactive protein (CRP). A dressing was applied over the laceration and the arm rested in a sling. The patient was admitted for observation and prophylactic antibiotics. Swab taken from the wound was negative for any microorganisms and the patient was discharged after 48 hours.

She was reviewed in the clinic at weekly interval. At the last review, three weeks after discharge, there was complete healing of the laceration with resolution of the subcutaneous emphysema.

## Discussion

Benign subcutaneous emphysema of the hand and upper limb of non-infectious aetiology has been documented in several case reports^1–4^. It is important to differentiate this condition from life threatening conditions like gas gangrene (clostridial myonecrosis) and necrotising fasciitis as the treatment and outcome differ considerably^1,3^. Table I outline the differentiating features.

**Table I:** Differentiating features of benign subcutaneous emphysema with gas gangrene or necrotising fasciitis

	Benign subcutaneous emphysema	Gas gangrene / Necrotising fasciitis
Onset	Following break in skin, most often trauma	No history of trauma
Development	Within 6-8 hours	About 12-18 hours
General condition	Well	Ill
Local signs	Usually absent	Features of infection
Blood Investigations	Within normal limits	Features of infection / sepsis
Emphysema on radiographs	Limited to subcutaneous tissue	+ involvement of deeper layers and muscle planes
Treatment	Mostly conservative / Supportive	Surgical, May need repeated debridement
Prognosis	Good, self-limiting	Progressive, life threatening

Benign subcutaneous emphysema has been reported to result from varied causes including migration of internal fixation devices, irrigation of wound with hydrogen peroxide, airgun, power or pneumatic tools and dental extraction^1,2,5^. It has also been reported after self-harm^4^, surgical procedures on hand^2^ and insect bite^3^.

Most authors agree that benign surgical emphysema results when air is sucked through a break in the skin which acts like a ‘ball valve type mechanism’^2,5^. Crampton proposed that application of bandage and mobilization creates a ‘ball-valve’ effect at the elbow resulting in extensive subcutaneous emphysema^4^.

It is agreed that most cases of benign subcutaneous emphysema can be treated by conservative measures when there is minimal pain and absence of systemic signs and extensive cellulitis^2^. Crampton recommended that the arm be immobilised after dressing to prevent a ‘ball-valve’ mechanism from occurring secondary to movement of the limb^4^. Subcutaneous air is absorbed by the body over one to three weeks depending upon the amount and site of emphysema.

Based on the information available in literature and experience of this case our suggestion for management of such case is i) Thorough history, examination and appropriate microbiological investigations are essential. ii) Wound care depends upon the size, site, contamination and presence of infection. Most puncture wounds around the joint less than 0.5 mm without contamination can be managed with dressing and a course of antibiotic according to local guideline. iii) It is important to immobilise the joint by arm sling or backslab to prevent a ‘ball-valve’ mechanism occurring around a hinge joint like elbow. iv) If the wound is contaminated, it needs proper debridement, irrigation, suturing, broad spectrum antibiotic in appropriate dose and duration and immobilization. v) The treating clinician should be aware of wound at and around the joint.

In the present case, certain typical features of benign subcutaneous emphysema were noted. The onset followed a small laceration and subcutaneous emphysema developed within 5-6 hours. The patient was systemically well with no local signs and normal blood counts and CRP. On the radiographs, the gas shadow was limited to the subcutaneous plane only.

## Conclusion

A careful history and identification of typical features can help in the diagnosis of benign subcutaneous emphysema. A timely diagnosis can help avoid unnecessary surgery as most cases can be treated by supportive measures. It also highlights the fact that not all cases of subcutaneous emphysema are due to gas gangrene.

## References

[1] De M, Stevenson J (2001). Subcutaneous emphysema of upper limb. Emerg Med J.

[2] Fox A, Sheick H, Ekwobi C, Ho-Asjoe M (2007). Benign surgical emphysema of the hand and upper limb: gas is not always gangrene - a report of two cases. Emerg Med J.

[3] Onwochei VE, Kelly ME, Lyons R, Khan W, Barry KM (2015). Benign subcutaneous emphysema: A case report with bite. Int J Surg Case Rep.

[4] Crampton JA (2005). An unusual case of surgical emphysema. Injury Extra.

[5] van der Molen AB, Birndorf M, Dzwierzynski WW, Sanger JR (1999). Subcutaneous tissue emphysema of the hand secondary to non-infectious etiology: a report of two cases. J Hand Surg Am.

